# *Heracleum persicum* L. extract protects gentamicin-induced testicular toxicity 

**DOI:** 10.22038/AJP.2024.24347

**Published:** 2024

**Authors:** Elnaz Khordad, Mohsen Akbaribazm, Seyed Moein Hosseini

**Affiliations:** 1 *Department of Anatomy, Torbat Heidariyeh University of Medical Sciences, Torbat Heidariyeh, Iran*; 2 *Department of Basic Medical Sciences, Khoy University of Medical Sciences, Khoy, Iran*; 3 *Student Research Committee, Torbat Heydariyeh University of Medical Sciences, Torbat Heydariyeh, Iran*

**Keywords:** Apoptosis, Male fertility, Antioxidant, Gentamicin, Heracleum persicum L.

## Abstract

**Objective:**

The objective of this study was to assess the protective effects of *Heracleum persicum* L. leaves extract (HPE) against oxidative damage induced by gentamicin (GM) in the testes of rats through biochemical, histopathological, and immunohistochemical approaches.

**Materials and Methods:**

Thirty-six male Wistar rats were divided into six groups (n=6/group) for 50 days. On day 51, the study assessed serum levels of luteinizing hormone (LH), follicle-stimulating hormone (FSH), and testosterone (TT), as well as antioxidant enzyme activity, nitric oxide levels, and various parameters related to testicular tissue (including the ferric reducing ability of plasma (FRAP), thiol, and thiobarbituric acid reactive substances (TBARS) levels). The stereological indices of seminiferous tubules were measured using serial sections of testicular tissue stained with hematoxylin and eosin, while the apoptosis rate of testicular parenchymal cells (p53, Caspase-3, and Bcl-2 positive cells) was also determined.

**Results:**

In the groups treated with HPE, particularly at 750 mg/kg, there was a significant increase (p<0.05) in LH, FSH, and TT hormone levels, an enhanced serum antioxidant enzyme activity and significantly reduced (p<0.05) nitric oxide levels. HPE inhibited the apoptotic pathway involving Bax/p53/Caspase-3 (significantly decreased (p<0.05) all three genes), thereby preserving the structure and function of the testicular tissue. Consequently, the number of p53 and Caspase-3 positive testicular cells decreased significantly (p<0.05), while the number of Bcl-2 positive cells increased.

**Conclusion:**

HPE demonstrated potential in protecting the function and structure of testis against toxic and oxidative damages.

## Introduction

Male infertility is a clinical condition that brings forth various physical, familial, social, and psychological complications. Research indicates that 15% of couples in the United States experience this disorder, while 25% of couples encounter sexual dysfunction linked to male infertility (Vander Borght and Wyns, 2018). The testicles play a crucial role in male fertility as they are responsible for producing testosterone (TT) and facilitating spermatogenesis. Injuries to the seminiferous tubules which comprise thirty percent of infertility cases in men, can result in compromised sperm quality and ultimately lead to infertility (Skakkebaek et al., 2016). 

Aminoglycoside antibiotics, including gentamicin (GM), are known to induce testicular toxicity by promoting the production of reactive oxygen spices (ROS) primarily through activation of the NF-κB/p38MAPK signaling pathway (Jiang et al., 2017). Within the testicles, GM disrupts the function of enzymes and mediators involved in scavenging free radicals, such as catalase (CAT), superoxide dismutase (SOD), glutathione (GSH), and glutathione peroxidase (GPx). This antibiotic leads to cellular deformation and shrinkage, mitochondrial swelling, lysosomal enlargement, and detachment of the spermatogenic lineage within the seminiferous tubules. Consequently, it often results in reduced testicle size, sperm count, and sperm motility (Aly and Hassan, 2018). Bukhari et al. (2011) demonstrated that prolonged exposure to non-therapeutic doses of GM can exacerbate atrophy and diminish germinal cells in the testicles, while also causing alterations in serum levels of luteinizing hormone (LH), follicle-stimulating hormone (FSH), and TT (Bukhari et al., 2022). GM also diminishes sperm motility by inhibiting the activity of Na/K ATPase ion pumps located in the flagellum of sperm, thereby interfering with their ability to move effectively. GM has been found to increase lipid peroxidation in the spermatogenic lineage, and Sertoli and Leydig cells (Naghdi et al., 2016).

Medicinal plants play a significant role in various stages of sperm proliferation and differentiation, impacting germ cells as well as enhancing the function of Leydig and Sertoli cells. They also contribute to maintaining the integrity of the blood-testis barrier and aiding in the clearance of toxic metabolites from the testicles (Mohammadi et al., 2013). When it comes to mitigating the damages caused by ROS induced by aminoglycosides, the use of antioxidants emerges as a crucial solution. Medicinal plants rich in polyphenolic compounds serve as important sources of antioxidants. These compounds have demonstrated synergistic effects with the body's own antioxidant systems, effectively protecting cells against the cytotoxic effects of ROS. Additionally, certain plants containing aphrodisiac compounds have shown efficacy in boosting the hypothalamic-pituitary-gonadal axis (HPGA), leading to increased testosterone concentration and ultimately enhancing libido, erection, and ejaculation functions, as well as promoting prostate secretion (Dutta and Sengupta, 2018). 

*Heracleum persicum* L., commonly referred to as “*golpar*” or Persian hogweed, is a medicinal plant belonging to the Apiaceae family. It is native to the Middle East, Eastern Europe, East and Southeast Asia and it has been traditionally used to treat various disorders, including Alzheimer's disease, polycystic ovary syndrome, hepatic-renal failure, and diabetes (Majidi and Lamardi, 2018; Asgarpanah et al., 2012). The hydroalcoholic extract of HPE leaves has demonstrated the ability to suppress the expression of cyclooxygenase-2 induced by interleukin 1β (IL-1β). Additionally, due to the presence of aphrodisiac compounds like tribulusterin and protodioscin, this plant may not only protect testicular tissue from oxidative and inflammatory damage but also affect enhance libido, TT secretion, and ultimately improve fertility (Akbaribazm et al., 2021). The objective of this study was to evaluate the protective effects of *Heracleum persicum* L. extract (HPE) against oxidative damage induced by gentamicin (GM) in the testes of rats. This assessment involved investigating the modulation of the HPGA, anti-oxidative mechanisms, and anti-apoptosis pathways. 

**Figure 1:**
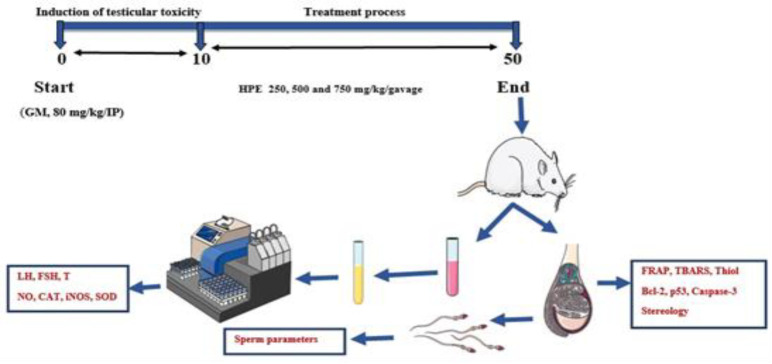
Research time line and experimental process

**Figure 2:**
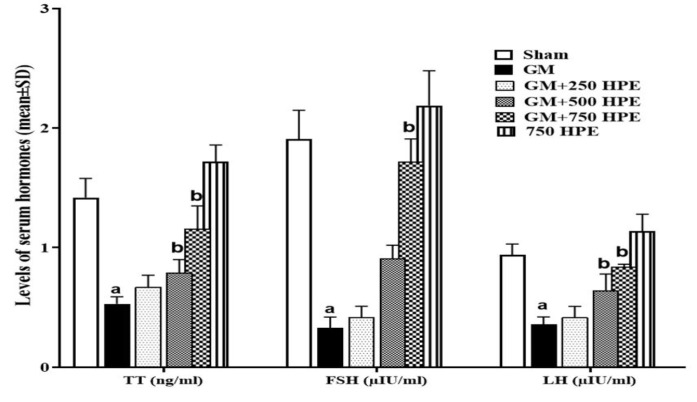
Rats’ body, epididymis, and testicular weights (g) in sham, GM, 250, 500 and 750 mg/kg HPE + GM (GM+ 250, 500 and 750 HPE) and 750 mg/kg HPE (750 HPE) treated groups (n=6/group; means±SD). ^a ^(p<0.05) sham vs. GM groups and ^b^ (p<0.05) GM vs. HPE treated groups.

**Figure 3:**
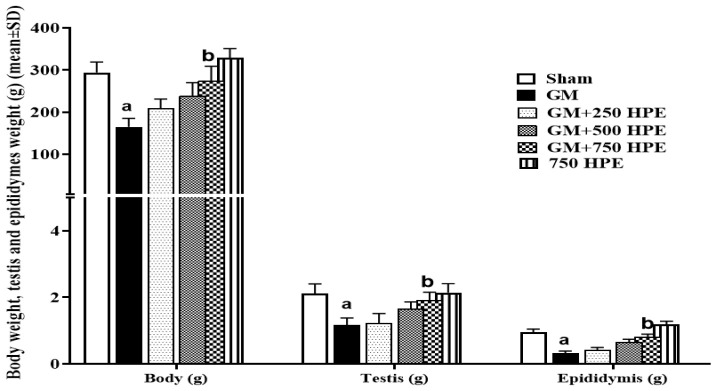
Serum levels of LH, TT, and FSH levels in sham, GM, 250, 500 and 750 mg/kg HPE + GM (GM+ 250, 500 and 750 HPE) and 750 mg/kg HPE (750 HPE) treated groups (n=6/group; means±SD). a (p<0.05) sham vs. GM groups and b (p<0.05) GM vs. HPE treated groups.

**Figure 4:**
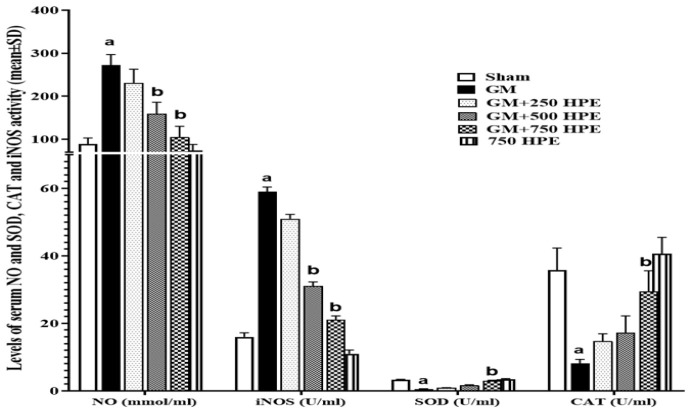
Serum activity of iNOS, CAT and SOD (U/ml) and also NO (mmol/ml) levels in sham, GM, 250, 500 and 750 mg/kg HPE + GM (GM+ 250, 500 and 750 HPE) and 750 mg/kg HPE (750 HPE) treated groups (n=6/group; means±SD). a (p<0.05) sham vs. GM groups and b (p<0.05) GM vs. HPE treated groups.

**Figure 5:**
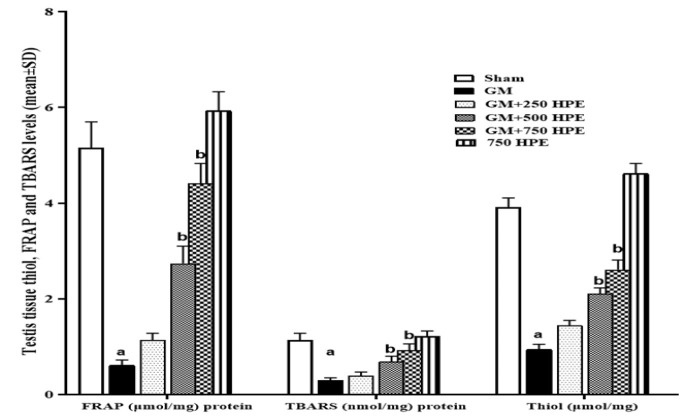
Testis tissue TBARS (nmol/mg proteins), thiol, and FRAP (µmol/mg protein) levels in sham, GM, 250, 500 and 750 mg/kg HPE + GM (GM+ 250, 500 and 750 HPE) and 750 mg/kg HPE (750 HPE) treated groups (n=6/group; means±SD). a (p<0.05) sham vs. GM groups and b (p<0.05) GM vs. HPE treated groups.

**Figure 6:**
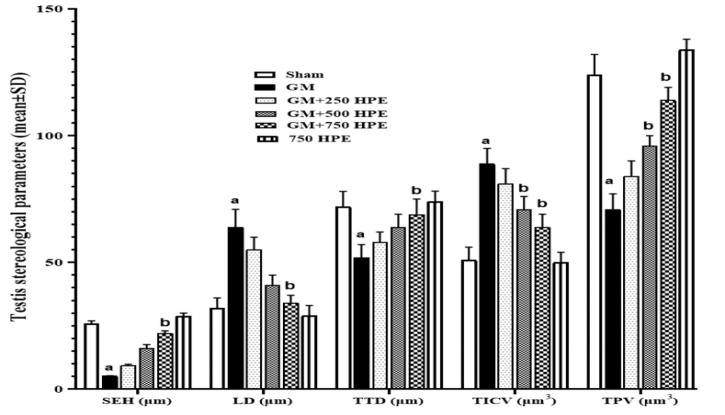
Histomorphometry and stereological assessments of testis tissue (SEH, LD, TTD, TICV, and TPV) in sham, GM, 250, 500 and 750 mg/kg HPE + GM (GM+ 250, 500 and 750 HPE) and 750 mg/kg HPE (750 HPE) treated groups (n=6/group; means±SD). a (p<0.05) sham vs. GM groups and b (p<0.05) GM vs. HPE treated groups.

**Figure 7:**
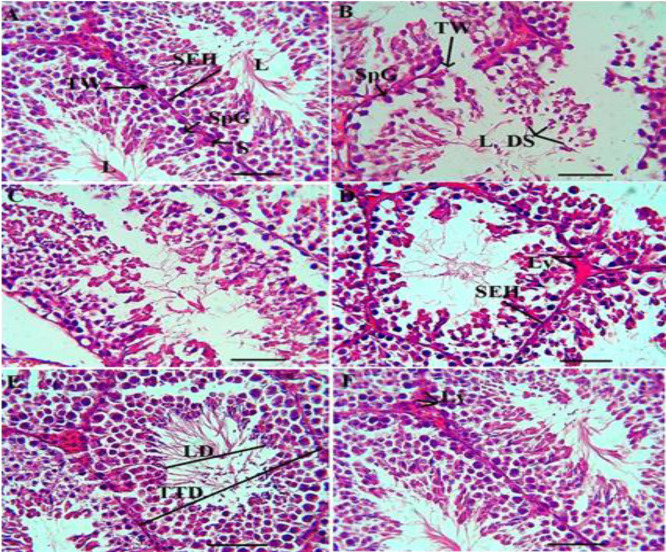
Histopathological changes of testis tissue in sham (A), GM (B), 250 (C), 500 (D) and 750 (E) mg/kg HPE + GM (GM+ 250, 500 and 750 HPE) and 750 (F) mg/kg HPE (750 HPE) treated groups. Sertoli cells (S), seminiferous epithelium height (SEH), tubular lumen (L), seminiferous tubular wall (TW), spermatogonia cells (SpG), Leydig cell (Ly), and degenerated spermatids (DS).

**Figure 8:**
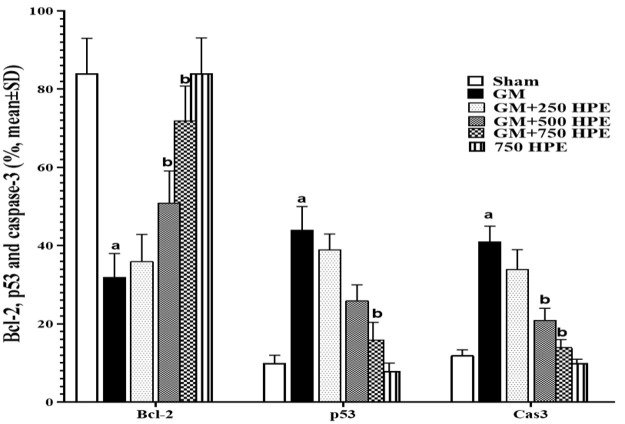
Immunohistochemistry of p53, Bcl-2, and Caspase-3 positive cells (%) in sham, GM, 250, 500 and 750 mg/kg HPE + GM (GM+ 250, 500 and 750 HPE) and 750 mg/kg HPE (750 HPE) treated groups (n=6/group; means±SD). ^a ^(p<0.05) sham vs. GM groups and ^b^ (p<0.05) GM vs. HPE treated groups.

**Figure 9:**
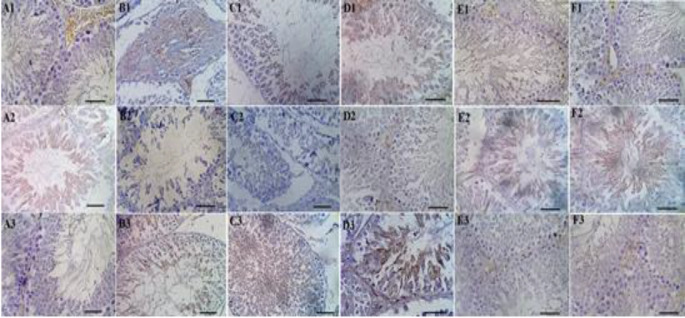
Immunohistochemistry [p53 (A1-F1), Bcl-2 (A2-F2) and Caspase-3 (A3-F3) positive cells, Scale bar = 50 μm, DAB staining × 400) in sham (A1-A3), GM (B1-B3), 250 (C1-C3), 500 (D1-D3) and 750 (E1-E3) mg/kg HPE + GM (GM+ 250, 500 and 750 HPE) and 750 (F1-F3) mg/kg HPE (750 HPE) treated groups.

## Materials and Methods

### HPE preparation

Fresh leaves of HP were collected from the rivers of Hamedan. After obtaining approval from a botanist, 5000 g of the leaves was dried in a dark place at 35±5°C. Subsequently, the dried leaves were ground using a grinder (model number: SA-45F, GlobalGilson company, US). The ground leaves were then dissolved in a mixture of 70% ethanol and distilled water (DW). After 72 hr, the resulting mixture was filtered using a filter paper (Whatman #42; Millipore, United States). The filtrate was further concentrated using a rotary evaporator (Asynt Ltd. Headquarters, United Kingdom), and the condensed mixture was dried at 40±5°C. Finally, 700 g of the resulting HPE was stored at 4°C until ready for use (Akbaribazm et al., 2020a). 

### Animals and study design

This study involved the use of thirty-six adult male Wistar rats, aged 90 days and weighing approximately 200±20 g. After a 72-hour adaptation period, the rats were housed in propylene cages under controlled conditions pf temperature 22±5°C, relative humidity 40±5%, and a 12/12 dark/light cycle. The rats were maintained in accordance with the animal care protocol and principles of the laboratory, under the supervision of the Torbat Heydarieh University of Medical Sciences (approval number: IR.THUMS.AEC.1401.008). They were provided with free access to standard rat pellets and water. During this period, animals had unrestricted access to standard rat pellets. Each 100 mg pellet is comprised of 10 Mcal energy, with 40 g of carbohydrates, 30 g of protein, and 30 g of total fat. Additionally, it contains 0.4 g of magnesium, 0.8 g of phosphorus, 1.6 g of calcium, 0.4 g of cobalt, 0.8 g of sodium, 0.2 g of iron as well as 150 mg each of arachidonic acid, palmitic acid, and linoleic acid. 

For the study, the rats were divided into six groups, with six rats in each group. The group allocation was as follows: Sham group: Rats received 0.1 ml (100 µl) of distilled water (DW) per day via gavage for 40 days.

Gentamicin group (GM): Rats received 80 mg/kg GM intraperitoneally (i.p.) for 10 days. GM and HPE co-treatment groups (GM+250, 500, and 750 HPE): Rats received 80 mg/kg GM (i.p.) injection for 10 days and 250, 500 and 750 mg/kg HPE via gavage for 40 days. HPE treatment group (750 HPE): Rats received 750 mg/kg HPE via gavage for 40 days. 

Throughout the study, GM was administered i.p. for 10 consecutive days, while HPE was administered orally for 40 consecutive days. The administration of GM and HPE occurred at specific times of the day, with GM given at 9 am and HPE given at 3 pm. To determine the appropriate dosage that would be effective yet non-toxic, various factors were taken into account. This included considering the LD_50_ (median lethal dose), conducting a pilot study, and reviewing previous research findings (research time line and experimental process are presented in Figure 1) (Asgarpanah et al., 2012; Akbaribazm et al., 2021). 

### Acute toxicity test (LD50)

To determine the LD_50_ of HPE, the modified two-step Lork's method was employed. In the first stage, three groups of rats (n=3 rats/group) were injected i.p. with doses of 25, 250, and 2500 mg/kg of HPE. Subsequently, in the next stage, three additional groups of rats (n=1 rat/group) were injected i.p. with doses of 50, 500, and 5000 mg/kg of the extract. The rats in both stages were closely monitored for 24 hr, assessing any signs of toxicity such as nausea, anorexia, depression, diarrhea, and potential mortality.

Based on the observed responses, the LD_50_ of HPE was calculated using the following formula: 

LD_50_ = (D_h_ × D_l_)^1/2^, 

where: D_h_ represents the highest dose of HPE extract that did not result in any deaths or toxic symptoms. D_l_ represents the lowest lethal dose of HPE extract at which deaths or toxic symptoms were observed in the rats (Lorke, 1983; Feng et al., 2022).

### Follicle-stimulating hormone (FSH), luteinizing hormone (LH), and testosterone (TT) levels

At the end of the study (the 51^st^ day), the rats were euthanized after undergoing pre-anesthesia (xylazine 2%; 80 mg/kg/i.p. injection) and anesthesia (ketamine 10%; 40 mg/kg/i.p. injection) procedures. Blood samples were gathered from the heart and then centrifuged at 12,000 g for 20 min to separate the serum. The serum hormone levels, including TT (Cat. No. 80552; Crystal Chem Biotech Company, United States), LH (Cat. No. 80969; Crystal Chem Biotech Company, United States), and FSH (Cat. No. CSB-E06871; Cusabio Biotech Company, United States), were measured using commercially available ELISA kits. The measurement process involved a colorimetric assay, following the manufacturer's recommendations and protocol (Akbaribazm et al., 2020b).

### Nitric oxide (NO) levels

Nitric oxide (NO) plays various biological roles at the physiological level, including vasodilation, cellular communication and regulation of immune Serum levels of nitric oxide (NO) were determined using a commercially available kit obtained from ZellBio (Cat. No. ZX-44107-96; ZellBio GmbH, Germany). The experimental procedure adhered to the manufacturer's instructions (Akbaribazm et al., 2020b).

### Catalase (CAT), inducible nitric oxide synthase (iNOS), and superoxide dismutase (SOD) activity

A commercially available ELISA kit, which employs a quantitative/colorimetric assay, and follows the manufacturer's protocol, was utilized to assess the serum activity of superoxide dismutase (SOD) (Catalog No. ab65354;), inducible nitric oxide synthase (iNOS) (Cat. No. ab126286), and CAT (Catalog No. ab277396, Abcam, Cambridge, MA, United States). The experimental procedure adhered to the manufacturer's instructions (Akbaribazm et al., 2020b).

### FRAP assay

To assess the overall antioxidant capacity of testis tissue using the FRAP method, the testis was dissected, and a homogenous tissue mixture was prepared. A total of 200 µl of the homogenous tissue was then combined with a FRAP solution comprising 5 ml of 2, 4, 6-Tripyridyl-riazine (TPTZ), 3 ml of ferric chloride, and 50 ml of acetate buffer. Subsequently, the mixture was incubated (at 37°C for 10 min). The absorbance of the resulting mixture was measured at a wavelength of 593 nm using a Shimazu Multispect spectrophotometer (model number: 1501, Shimadzu, Japan). The total antioxidant capacity is expressed as µmol/mg protein (Akbaribazm et al., 2020b).

### Lipid peroxidation levels (TBARS)

The measurement of lipid peroxidation levels in testicular tissue was conducted using the thiobarbituric acid reactive substances (TBARS) method. In this approach, a TBARS reaction solution was prepared, consisting of 50 μl phosphoric acid, 50 μl thiobarbituric acid, and 2 μl butylated hydroxytoluene. The reaction solution was then added to the 50 μl homogenized testicular tissue. Following an incubation period of 20 min at 60°C and subsequent centrifugation (12,000 g for 5 min), the absorbance of the supernatant was measured at 532 nm using a Mettler-Toledo spectrophotometer (model number: UV5, Indonesia). The results are reported in nmol/mg (Akbaribazm et al., 2020b).

### Immunohistochemistry (IHC) analysis

Caspase-3, p53, and Bcl-2 were employed as markers for apoptotic-differentiation in the spermatogenic lineage. The testis tissues were washed with phosphate buffered saline (PBS) and underwent standard tissue processing, resulting in the preparation of paraffin blocks from the tumor. Then, 5 µm sections were placed on slides and incubated overnight at 95°C and then for 1 hr at 25°C with primary antibodies against Bcl-2 (1:1000; Cat. No. sc-7382), Caspase-3 (1:1000; Cat. No. CPP324-1-18/ sc-56052), and p53 (1:1000; Cat. No. Pab 1801/sc-98) obtained from Santa Cruz Biotechnology, Inc. (US). Washing buffer containing Tween-20 was used for rinsing, and bovine serum albumin (5%) was utilized to block residual antibodies. Subsequently, the slides were treated with 3% hydrogen peroxide (H_2_O_2_) at 25°C for 20 min and then with 3,3'-diaminobenzidine (DAB). Hematoxylin was employed as a counterstain for all slides. An optical Olympus microscope (model number: IX71 microscope, Japan) connected to Moticam (Technologies, Japan) camera system was employed to examine the slides at a magnification of 100X. The percentage (%) of Caspase-3, Bcl-2, and p53 positive cells out of the total cells was determined by analyzing 10 random fields of view in each sample (Akbaribazm et al., 2020a).

### Histopathology and stereology of testis tissue 

The method begins with testis samples immersed in 10% formalin for 72 hr to preserve them. Initial and final volumes of structures were determined using immersion and Cavalieri principle methods, respectively, considering tissue shrinkage. Isotropic uniform random (IUR) slices were prepared using the Orientator method, involving random cuts into 2 mm sections. From each tissue, 6-8 sections were randomly selected, and a circular piece was taken for measurement. Sections underwent tissue processing and embedding in paraffin before being sliced into 5 µm sections using a rotary microtome. Tissue shrinkage was determined using the area of punched samples before and after processing. The relative volume of testicular structures [testis parenchymal volume (TPV) and testis interstitial volume (TICV)] was calculated using a point probe method on a grid, and the final volume of each structure was determined by multiplying the relative volume by the reference volume. Seminiferous epithelium height (SEH), lumen diameter (LD), and total tubular diameter (TTD) were measured in 10 random fields of view per slice stained with hematoxylin and eosin, using an Olympus microscope (model number: IX71 microscope, Japan) connected to Moticam (Technologies, Japan) camera system. Morphometric parameters are analyzed using Image J software. This comprehensive method ensures accurate measurement and analysis of testicular structures and morphometric parameters (Akbari et al., 2017). 

### Statistical analysis

The data are presented as mean±standard deviation (SD). Graphs were generated using GraphPad Prism version 8 software, and statistical analysis was conducted using SPSS version 16 (ver. 16, IBM Corp.). The normality of the data was assessed using the Kolmogorov-Smirnov test (p>0.05). To determine differences in variance among the data, a one-way analysis of variance (ANOVA) was performed, followed by Tukey's *post hoc* descriptive test. A p<0.05 was considered statistically significant.

## Results

### Acute toxicity test (LD50)

After conducting a 24-hr monitoring of the groups, the LD_50_ of HPE was determined. It was observed that the highest dose (D_h_) at which no deaths or toxic symptoms occurred in rats, was 2500 mg/kg, while the lowest dose (D_l_) at which deaths and toxic symptoms were observed was 5000 mg/kg. By applying Lork's formula, the LD_50_ of HPE was calculated to be 3535 mg/kg (equivalent to 3.535 g/kg). These findings suggest that lower doses can be utilized in animal studies.

### Rats’ testicular, body, and epididymis weights

The administration of GM resulted in a significant reduction (p<0.05) in all three weight parameters compared to the sham group. However, treatment with HPE showed a dose-dependent improvement in weight parameters in rats. Specifically, the increase in weight observed in the group treated with 750 mg/kg HPE (GM+750 HPE) was statistically significant (p<0.05) when compared to the GM group (Figure 2).

### Serum levels LH, TT, and FSH

GM, through its impact on the HPGA, exerted suppressive effects, leading to a significant decrease (p<0.05) in all three hormonal parameters compared to the sham group. In contrast, HPE, possibly due to its polyphenolic and aphrodisiac compounds, demonstrated the ability to enhance the HPGA function, resulting in a dose-dependent increase in the levels of all three hormones. Specifically, the levels of TT and luteinizing hormone (LH) showed a significant increase (p<0.05) at doses of 500 and 750 mg/kg HPE compared to the GM group. Additionally, the increase in follicle-stimulating hormone (FSH) level compared to GM was significant (p<0.05) only in the 750 mg/kg HPE (GM+750 HPE) group (Figure 3).

### Serum activity of iNOS, SOD, and CAT and NO levels

GM and its metabolites were found to induce the production of free radicals, leading to a significant decrease (p<0.05) in the activity of endogenous antioxidant enzymes, including iNOS, SOD, and CAT. This decrease in antioxidant capacity resulted in increased levels of nitric oxide (NO) compared to the sham group. However, treatment with HPE demonstrated a dose-dependent effect in enhancing the activity of endogenous antioxidant enzymes. Consequently, the level of NO decreased significantly (p<0.05) in the 750 mg/kg HPE (GM+750 HPE) group compared to the GM group, while the activity of all three enzymes showed a significant increase (p<0.05). Notably, in the 500 mg/kg HPE (GM+750 HPE) group, a significant (p<0.05) decrease in NO levels and a significant (p<0.05) increase in iNOS activity were also observed compared to the GM group (Figure 4).

### Testis tissue levels of TBARS, thiol, and FRAP

The assessment of testicular tissue's total antioxidant capacity and lipid peroxidation in different groups revealed that GM significantly (p<0.05) reduced the levels of all three indicators, namely TBARS, thiol, and FRAP, compared to the sham group. However, treatment with HPE exhibited a dose-dependent inhibition of lipid peroxidation by enhancing the total antioxidant capacity. Consequently, HPE administration led to increased levels of all three antioxidant indices, namely TBARS, thiol, and FRAP, when compared to the GM group. Statistical analysis demonstrated that the observed increase in all three indicators in the GM+500 and GM+750 HPE groups (at doses of 500 and 750 mg/kg HPE, respectively) was significant (p<0.05) compared to the GM group (Figure 5).

### Testis tissue stereological (seminiferous epithelium height (SEH), lumen diameter (LD), total tubular diameter (TTD), testis parenchymal volume (TPV), and testis interstitial volume (TICV)) parameters

 GM administration resulted in notable alterations in testicular stereological parameters, including damage to the generative parenchyma, destruction and shrinkage of seminiferous tubules, induction of apoptosis in the spermatogenic lineage, disruption of the seminiferous tubule walls, hypertrophy, congestion, and interstitial edema. Specifically, GM significantly (p<0.05) decreased the indices of SEH, TTD, and TPV compared to the sham group. Conversely, it significantly (p<0.05) increased the indices of LD and TICV.

In contrast, treatment with HPE preserved tubule membrane integrity in a dose-dependent manner, restored the normal arrangement of the germinal epithelium, and exhibited cyclic visibility of the spermatogenic lineage within the tubules. Furthermore, HPE prevented interstitial congestion and edema by restoring a normal vascular system. Stereological analysis revealed that HPE at doses of 500 and 750 mg/kg (in the GM+500 and GM+750 HPE groups) significantly (p<0.05) increased the indices of parenchymal tubules (SEH, TTD, and TPV) and significantly (p<0.05) decreased the indicators of tubular and interstitial damage (LD and TICV) compared to GM (Figures 6 and 7).

### Bcl-2, p53, and Caspase-3 positive cells in testicular tissue

GM administration resulted in a significant (p<0.05) increase in the percentage of p53 and Caspase-3 positive cells, while causing a significant (p<0.05) decrease in the percentage of Bcl-2 positive cells in testicular tissue. These effects were attributed to the strengthening of the mitochondrial apoptotic cascade when compared to the sham group. However, treatment with HPE exhibited protective effects by inhibiting the mitochondrial intrinsic apoptotic pathways in a dose-dependent manner. Specifically, HPE at doses of 500 and 750 mg/kg (in the GM+500 and GM+750 HPE groups) significantly (p<0.05) decreased the percentage of p53 and Caspase-3 positive cells, while significantly (p<0.05) increasing the percentage of Bcl-2 positive cells compared to GM (Figures 8 and 9).

## Discussion

### GM induced testicular toxicity

The findings of this study reveal that GM induces testicular toxicity by inhibiting the HPG axis and activating apoptotic, inflammatory, and antioxidant pathways.

Investigations into the impact of GM exposure on testes and sperm parameters have been conducted in animal models. These studies indicate that GM can induce testicular toxicity, adversely affecting various sperm parameters. Rat studies, for instance, demonstrate that exposure to GM results in diminished sperm count, motility, and viability, accompanied by abnormalities in sperm cell structure (Elsawah et al., 2022). Furthermore, exposure to GM has been found to instigate oxidative stress and disrupt antioxidant defense mechanisms in the testes, leading to sperm damage. In mouse studies, GM exposure has been associated with testicular toxicity, characterized by seminiferous tubule degeneration, reduced sperm production, and alterations in sperm morphology (Yahya et al., 2019).

The study also brought attention to potential disturbances in hormonal regulation within the testes induced by GM. Various research findings suggest that GM has the ability to generate ROS and free radicals, including superoxide anions and hydroxyl radicals. These ROS and free radicals can instigate oxidative stress, leading to damage in essential cellular components such as, deoxyribonucleic acid (DNA), proteins, and lipids. Moreover, GM possesses the capability to activate several signaling pathways by releasing free radicals, including NF-κB, mitogen-activated protein kinases (MAPKs), the p53 pathway, and the phosphatidylinositol 3-kinase (PI3K)/Akt pathway (Ali et al., 2020).

Aly (2019), in an assessment of the impact of GM toxicity on mouse testes, found that this antibiotic elevates apoptotic pathways, as evidenced by an increased expression of Caspase-3 and -9 in testicular parenchymal tissue. The study also observed that GM metabolites led to a reduction in serum SOD, CAT, glutathione peroxidase, and glutathione reductase activities (Aly, 2019). Oxidative stress induced by GM triggered both death receptor and mitochondrial apoptotic pathways. The current study's results further indicated that GM increased the expression of iNOS and NO levels, accompanied by a decrease in the activities of SOD and CAT. Additionally, GM was found to reduce the total antioxidant capacity while elevating lipid peroxidation levels. These oxidative changes promoted apoptosis, ultimately increasing the number of Caspase-3 positive cells and decreasing Bcl-2 positive cells. In a separate investigation, Hamoud (2019) demonstrated that GM heightened the count of Caspase-3 and Ki-67 cells by elevating levels of free radicals. This study provided evidence that GM stimulates apoptotic pathways in spermatogenic cells (Hamoud, 2019).

### HPE ameliorates GM-induced testicular toxicity

The present investigation provides compelling evidence that HPE exhibits a dose-dependent amelioration of GM-induced testicular toxicity, particularly at a dosage of 750 mg/kg. This beneficial impact is mediated through diverse mechanisms. HPE contains a range of polyphenolic compounds, notably flavonoids, which exert multifaceted effects. As demonstrated by Roshanaei et al. (2017), the plant extract exhibits protective properties against liver damage induced by carbon tetrachloride (CCl4) toxicity. Their findings indicate that the plant extract enhances the activity of CAT, SOD, peroxidase (POD), glutathione reductase (GSR), and glutathione S-transferase (GST) in the liver, signifying reinforced antioxidant defenses. Moreover, it enhances the total antioxidant capacity and raises tissue TBARS levels in the liver, thereby preserving the liver's functional and structural integrity in the face of CCl4 toxicity (Roshanaei et al., 2017). Numerous *in vivo* and *in vitro* studies underscore the protective attributes of HPE. In a research investigation focusing on the effects of HPE on chromatin quality and sperm parameters in mice, Taghizabet et al. (2016) unveiled positive outcomes associated with the plant extract. The findings indicated that the extract plays a beneficial role in maintaining sperm chromatin quality and enhancing various sperm parameters. Comparative analysis with the control group demonstrated elevated total sperm count, viability, and progressive sperm motility (Taghizabet et al., 2016). Furthermore, a study conducted by Akbaribazm et al. (2021) explored the impact of HPE on rats experiencing GM-induced nephrotoxicity, yielding intriguing results. The investigation revealed the anti-inflammatory, anti-oxidative, and anti-apoptotic properties of the plant extract. Notably, when administered at doses of 500 and 750 mg/kg, the extract demonstrated the capability to reduce cytokine levels. Additionally, it effectively alleviated systemic pro-inflammatory factors such as TNF-α, IL-1β, and IL-6. The plant extract exhibited inhibitory effects on the Bax/Bcl-2/p53/ Caspase-3 apoptotic pathway within the tissue of all rats receiving the extract, compared to the group administered with GM (Akbaribazm et al., 2021). 

In the course of liquid chromatography electrospray ionization tandem mass spectrometric (LC/ESI-MS/MS) analysis of HPE, various compounds were identified, including biochanin A-7-glucoside, p-coumaric acid, salicylic acid, gallic acid, kaempferol, quercetin, genistein, ferulic acid, daidzein, and aphrodisiac compounds. Particularly noteworthy is the presence of tribulusterin and protodioscin, suggesting potential effects on the HPG axis and serum testosterone levels (Majidi and Lamardi, 2018). Therefore, in addition to its well-established anti-inflammatory, anti-apoptotic, and potent antioxidant properties, this plant may also play a role in enhancing the HPG axis and regulating serum testosterone levels. Research indicates that plants containing tribulusterin and protodioscin can enhance spermatogenesis, influencing sperm count, motility, and morphology. These compounds are believed to stimulate Leydig cells, resulting in increased testosterone secretion through the HPG axis (Wang et al., 2021a). Additionally, they are thought to offer protection to Sertoli cells, fostering the differentiation of spermatogonial cells into spermatozoa (Wang et al., 2021b). Furthermore, various studies have demonstrated that genistein, quercetin, daidzein, and kaempferol can boost spermatogenesis and protect spermatogonia from damage. These effects are believed to be mediated through different molecular pathways, including ERK1/2, Nrf2/HO-1, NF-κB/p38MAPK, and PI3K/Akt/mTOR (Ma et al., 2019; Sun et al., 2022). Moreover, these compounds aid in preventing inflammation and oxidative stress. In a study by Dalouchi et al. (2014) on the protective effects of HPE during cyclophosphamide-induced toxicity in spermatogenesis, it was observed that the extract, known for its high polyphenolic content, significantly increased the total antioxidant capacity and the activity of endogenous antioxidant enzymes. Additionally, HPE was found to enhance the viability and motility of sperm under cyclophosphamide-induced testicular toxicity by increasing intracellular ATP levels and preserving the membrane integrity of spermatozoa (Dalouchi et al., 2014). In this current investigation, it was noted that HPE showcased the capacity to mitigate mitochondrial apoptosis through the enhancement of the HPG axis and the elevation of TT levels. Furthermore, HPE was observed to enhance the overall antioxidant capacity by boosting the levels of FRAP, thiol, and TBARS. Additionally, HPE exhibited inhibitory effects on inflammatory pathways by suppressing the expression of p53 and Caspase-3 in the testicular parenchyma.

The findings of this study underscore the protective potential of HPE against testicular damage induced by GM exposure. HPE demonstrated a multifaceted mechanism of action, bolstering the HPGA and activating endogenous antioxidant defenses against ROS. Moreover, it effectively maintained the structural integrity of testicular tissue and preserved stereological parameters crucial for reproductive function. Notably, HPE exhibited inhibitory effects on apoptotic pathways mediated by key regulators such as Bcl2, p53, and Caspase-3. The antioxidant properties of HPE were instrumental in safeguarding the functionality of critical nurse cells, including Sertoli and Leydig cells, while concurrently supporting the intricate process of spermatogenesis. Consequently, improvements in sperm parameters were observed despite the presence of ROS-induced injuries. These beneficial effects highlight the pivotal role of aphrodisiac and polyphenolic compounds inherent in HPE, which collectively contribute to the preservation of male fertility in the face of toxic insults. Overall, the study underscores the potential of HPE as a promising therapeutic agent in mitigating testicular damage induced by harmful substances, thus offering prospects for enhancing reproductive health in males.
